# Supplementary material for: A rapid, low pH, nutrient stress, assay to determine the bactericidal activity of compounds against non-replicating Mycobacterium tuberculosis
Source: PLoS One. 2019 Oct 7;14(10):e0222970. doi: 10.1371/journal.pone.0222970 (PMC6779252; doi:10.1371/journal.pone.0222970)
Supplement: S1 Fig — Briefly, a late log-phase culture of the luciferase-expressing strain of M. tuberculosis was adjusted to OD590 1.0 in PBS-Tyloxapol. Serial dilutions were prepared and RLU and CFU determined from triplicate wells. Data are mean +/- standard deviation. RLU = relative light units. CFU = colony forming units. (DOCX) [file pone.0222970.s001.docx]

Supporting Information

**S1 Fig.** We determined the CFU to RLU correlation in 4 independent experiments. Briefly, a late log-phase culture of the luciferase-expressing strain of *M. tuberculosis* was adjusted to OD_590_ 1.0 in PBS-Tyloxapol. Serial dilutions were prepared and RLU and CFU determined from triplicate wells. Data are mean +/- standard deviation. RLU = relative light units. CFU = colony forming units.
